# Evaluation of solvation free energies for small molecules with the AMOEBA polarizable force field

**DOI:** 10.1002/jcc.24500

**Published:** 2016-10-19

**Authors:** Noor Asidah Mohamed, Richard T. Bradshaw, Jonathan W. Essex

**Affiliations:** ^1^ Computational Systems Chemistry, School of ChemistryUniversity of SouthamptonHighfieldSouthamptonSO17 1BJUK

**Keywords:** solvation free energies, log P, molecular dynamics, polarizable force field, fixed‐charge force field

## Abstract

The effects of electronic polarization in biomolecular interactions will differ depending on the local dielectric constant of the environment, such as in solvent, DNA, proteins, and membranes. Here the performance of the AMOEBA polarizable force field is evaluated under nonaqueous conditions by calculating the solvation free energies of small molecules in four common organic solvents. Results are compared with experimental data and equivalent simulations performed with the GAFF pairwise‐additive force field. Although AMOEBA results give mean errors close to “chemical accuracy,” GAFF performs surprisingly well, with statistically significantly more accurate results than AMOEBA in some solvents. However, for both models, free energies calculated in chloroform show worst agreement to experiment and individual solutes are consistently poor performers, suggesting non‐potential‐specific errors also contribute to inaccuracy. Scope for the improvement of both potentials remains limited by the lack of high quality experimental data across multiple solvents, particularly those of high dielectric constant. © 2016 The Authors. Journal of Computational Chemistry Published by Wiley Periodicals, Inc.

## Introduction

Much effort has been devoted to advancing computational techniques to predict free energies in biomolecular systems, ranging from more theoretically rigorous (e.g., alchemical free energy calculations) to less rigorous (e.g., continuum solvation, docking and scoring) methods.^1^ As with any computational approach, accuracy in predicting experiment requires a synergy of sufficient conformational sampling with an accurate molecular mechanics potential energy function describing the intermolecular interactions.^2^ Many sampling issues have been dealt with using intensive enhanced sampling methods coupled to molecular dynamics (MD)^3^, ^4^, ^5^ or Monte Carlo methods.^6^, ^7^ However, the issues associated with potential energy function or force field accuracy are substantially more problematic and remain a major challenge in force field development and molecular recognition applications.^8^, ^9^

Within the range of fixed‐point‐charge, pairwise additive MM force fields available for molecular simulation,^8^, ^10^, ^11^, ^12^, ^13^, ^14^, ^15^, ^16^, ^17^ a number of philosophies exist for the derivation of atomic partial charges and calculation of electrostatic interactions. These models often take account of polarization implicitly in the derivation of charges, and are mainly parameterized to recreate interactions in the aqueous phase. This limits their ability to fully adapt to changes in environment. To improve the accuracy of interatomic potentials for biomolecular interactions, the Atomic Multipole Optimized Energetics for Biomolecular Application (AMOEBA) force field has been introduced.^18^ AMOEBA is an advanced potential energy function including a polarizable molecular mechanics model,^18^, ^19^, ^20^ designed to directly treat polarization effects by incorporating an explicit response of induced atomic dipoles to the instantaneous molecular environment. The ability of the AMOEBA force field to capture these effects may be expected to result in parameters with greater transferability than standard fixed‐point‐charge models, and thereby give accurate predictions of interaction energetics across a variety of systems.

Consequently, an evaluation of potential energy function accuracy is needed to determine the quality of their performance, particularly given the added computational cost of explicit polarizable potentials. Commonly, solvation free energy calculation approaches^20^, ^21^, ^22^, ^23^, ^24^, ^25^, ^26^ have been performed to assess force field properties. This is thanks to the availability of high accuracy experimental data, and the straightforward computational methodologies for free energy prediction. As such, evaluating the accuracy of solvation free energy prediction is often a crucial step for force field validation.

Water has been used as the solvent to assess the accuracy of physical models in solvation free energy approaches (as opposed to organic solvents) in most studies,^23^, ^26^, ^27^, ^28^, ^29^, ^30^, ^31^ due to the extensive experimental data available for the interaction between a solute and water and its significant biological relevance. However, to investigate the effect of electronic polarization in biomolecular systems, solvation free energies in solvents other than water are worthy of consideration due to the changes in dielectric environment that may occur in a biomolecular situation, for example, the difference between a protein interface and bulk solvent, or between a membrane surface and the interior of a bilayer.

Compared to the extensive studies performed with water, there are comparatively few large‐scale studies of organic solvents. Recently, Caleman et al. evaluated the performance of GAFF^32^ and OPLS/AA^33^ in organic solvents.^34^ They benchmarked the force fields by computing liquid properties such as density, enthalpy of vaporization, heat capacity, surface tension, isothermal compressibility, volumetric expansion coefficient, and dielectric constant of ∼150 organic liquids. A more recent paper by Genheden has calculated solvation free energies for approximately 150 small organic molecules, derived from the Minnesota solvation database, using a simple all‐atom/coarse‐grained hybrid model (AA/ELBA). This study showed good agreement (<1.0 kcal mol^−1^) of solvation free energies with experiment, albeit in four related polar solvents and three related nonpolar solvents.^35^ In a larger study, Zhang et al. compared the performance of GAFF with three different prediction methodologies for solvation free energies (thermodynamic integration, a quantitative structure‐property relationship [QSPR] and the conductor‐like screening model for realistic solvation [COSMO‐RS]), employing a wide range of organic solvents.^36^ These studies involved the evaluation of Gibbs solvation free energies for 228 organic molecules in organic solvents compared against experimental data. Based on their analysis, no significant difference in correlation was shown between different prediction models with the GAFF force field. However, the authors also highlighted the fact that it is difficult for a fixed‐point‐charge force field such as GAFF to accurately reproduce both liquid properties and solvation properties simultaneously across a large number of solvents due to the absence of explicit electronic polarization to take into account changes in molecular environment.

To determine whether the explicit inclusion of polarization in a potential energy function is able to improve the accuracy of its free energy calculations over a much simpler and cheaper energy function, here we evaluate the performance of the AMOEBA model. Previously, AMOEBA performance has been tested for hydration free energy predictions,^20^, ^22^, ^37^, ^38^ but the additional computational cost of the AMOEBA potential over pairwise additive models has meant that large scale studies, and free energies in solvents other than water, have not traditionally been performed. In this paper, we evaluate AMOEBA performance by calculating the solvation free energies of a set of small molecule solutes across a range of four common organic solvents, giving a total of 54 solute‐solvent systems, each evaluated in triplicate. The test was carried out using solvents of different dielectric constants representing a variety of electrostatic environments to investigate the transferability of parameters between diverse systems. Manual parameterization was performed for each solute following the recommendations in a previous AMOEBA parameterization.^39^ Computational solvation free energies were then validated against experimental data. In addition, we also compare AMOEBA with solvation free energies generated using the GAFF fixed charge model, to measure any improvements arising by incorporating an explicit polarization term. Ultimately, statistical error analysis was carried out to validate the significance of observed differences between calculated solvation free energies for both force fields.

## Methods

### Dataset

**Figure 1:**
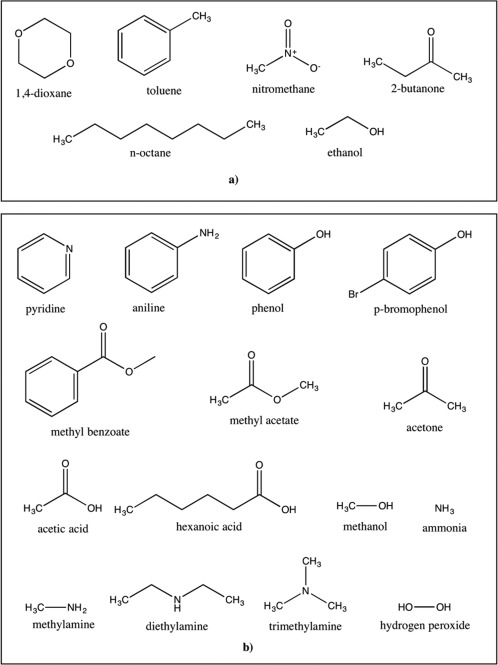
The structures of small molecules selected in this study. a) Dataset of small molecules for toluene, chloroform, acetonitrile and DMSO solvent. b) Dataset of additional small molecules for toluene and chloroform solvent.

A total of 21 small molecules (Fig. 1) with a variety of functional groups were selected in this study: six molecules had experimental solvation free energies for all four nonaqueous solvents (Fig. 1a), and 15 further molecules had experimental solvation free energies for only toluene and chloroform solvent (Fig. 1b). This choice of small molecules was taken from the Minnesota solvation database^40^ and Abraham et al., 1999.^41^ Although the Minnesota solvation database contains in excess of 3000 data points, our dataset for this study was limited to molecules for which (a) experimental solvation free energies were available in multiple organic solvents, and (b) these multiple organic solvents had parameters available in the amoeba09 or chloroalkane AMOEBA force fields.^39^, ^42^ Solvent models in both force fields have previously undergone limited validation including the calculation of liquid density and enthalpy of vaporization to assess their suitability.^39^, ^42^

### Nonaqueous solvents

Considering the availability of experimental solvation free energies for a variety of different molecules, four common organic solvents with a range of dielectric constants were chosen: toluene (*ε* = 2.38), chloroform (*ε* = 4.81), acetonitrile (*ε* = 36.64), and dimethylsulfoxide (DMSO, *ε* = 47.24).^43^ Here, all the AMOEBA solvent models were prepared using the parameters taken from amoeba09.prm^39^ except for chloroform.^42^ The most recent AMOEBA chloroform parameters published by Ren and coworkers,^42^ which made use of the ForceBalance parameter optimization protocol, were used.^44^ For fixed‐charge simulations, solvent parameters were taken from Cieplak et al. (chloroform),^45^ Grabuleda et al. (actetonitrile),^46^ and Dupradeau et al. (toluene and DMSO).^47^ For consistency, the setup of solvated systems was identical for both force fields, as explained in the solvent box preparation section below.

### Parameterization

Manual parameterization was performed to improve the consistency and accuracy of the small molecule parameters for AMOEBA. In manual parameterization, the parameters were generated by following the standard AMOEBA parameterization protocol^39^ defined by Ponder and coworkers, using the TINKER 6.3.3 package^48^ and GAUSSIAN09 program.^49^ Where valence parameters (bond, angle, stretch‐bend, out‐of‐plane, and torsion), van der Waals parameters and atomic polarizabilities for the small molecules were already available, they were taken directly from the TINKER amoeba09.prm force field.^39^ For small molecules that had not already been parameterized in amoeba09, the multipole coordinate frames and polarization groups were manually defined and the valence parameters assigned according to the suggested parameters using the TINKER valence program, refined by comparison with parameters for similar atom types in amoeba09. In all cases, atomic multipole parameters for molecules were derived from QM calculations performed with GAUSSIAN09^49^ using three steps.^50^ Essentially, the AMOEBA parameterization procedure requires only the initial coordinates of a molecule to assign the entire AMOEBA potential for that molecule. First, the initial structure of each molecule was optimized quantum mechanically at the HF/6‐31G* level using GAUSSIAN09.^49^ A single‐point energy calculation was carried out subsequently at the MP2/6‐311G(1d, 1p) level of theory followed by a Distributed Multipole Analysis facilitated by the Gaussian Distributed Multipole Analysis (GDMA) program^51^ of Stone to compute an initial set of atomic multipoles, using the original DMA procedure.^52^ This was continued by a further single point calculation of the molecular electrostatic potential using a larger basis set (MP2/aug‐cc‐pVTZ). Finally, the AMOEBA dipole and quadrupole parameters were optimized by fitting to the QM electrostatic potential from the latter single point calculation.

At the same time, the small molecules were also parameterized for the GAFF fixed‐point charge force field as a comparison. All the parameterization for the small molecules was performed following the standard GAFF fixed‐point‐charge parameterization procedures. The ANTECHAMBER program^53^ from the AMBER 14 package was used to derive the fixed‐point‐charge parameters of small molecules for the MD simulations, implementing AM1‐BCC atomic charges.^54^, ^55^ Generated parameters for all solutes are available freely as an online dataset.^56^

### Free energy calculations

**Figure 2:**
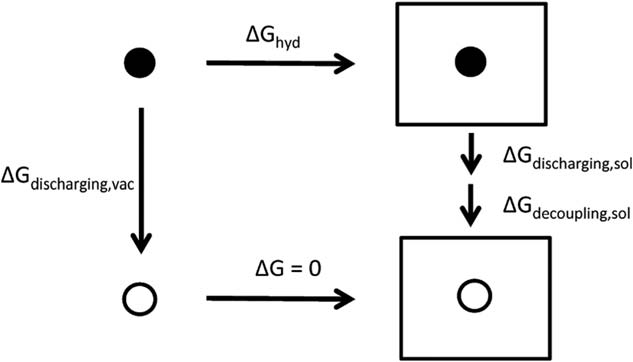
Thermodynamic cycle^37^ adopted for calculating the solvation free energy of small molecules in four different nonaqueous solvents. The simulations involve three sets of calculations run in vacuum and in solvent (square box). Black circles represent a fully charged solute interacting with its environment, while the circle with no fill denotes a discharged and completely decoupled system. The gas phase intermolecular interactions (vdW decoupling) do not need to be evaluated because there is no interaction between the solute and the environment in vacuum.

The solvation free energies of small molecules in four different solvents were calculated by adopting the protocol for hydration free energy calculations from Shi et al.^37^ The estimated solvation free energies of each molecule were computed based on the thermodynamic cycle (Fig. 2) for solvation free energy in explicit nonaqueous solvent molecular dynamics simulations. The overall solvation free energy is denoted by:

(1) $$\Delta G_{\text{solv}\;}=\;-\Delta G_{\text{decoupling},\text{sol}}\;-{\;\Delta G}_{\text{discharging},\text{sol}\;}+{\;\Delta G}_{\text{discharging},\text{vac}}$$

Three sets of calculations were required: (i) the discharging of molecule in solvent, (ii) the decoupling of van der Waals (vdW) interactions between solute and environment in solvent, and (iii) the discharging of solute in vacuum. For the evaluation of Δ*G*_solv_ for molecules in each solvent, MD simulations were run for both AMOEBA and GAFF force fields by applying a similar system setup. Finally, Bennett's Acceptance Ratio (BAR)^4^, ^57^ was used to compute free energy differences for each perturbation.

### Nonaqueous solvent box preparation

A cubic box of solvent with approximately ∼ 40 Å dimension on each side, containing ∼ 400 to 800 molecules, was first prepared for each solvent using TINKER utilities.^48^ The number of solvent molecules inserted in the box varied depending on the size of solvent molecule and the experimental density required. The solvent box was then minimized with the steepest descent algorithm for 2500 steps and heated to 300 K at constant volume using NVT MD over a 50 ps time period, followed by 200 ps equilibration to 1 atm at constant pressure in the NPT ensemble. A Berendsen barostat was applied to constrain the pressure with coupling time set at 2 ps.^58^ This simulation was run with 1 fs time steps using the Velocity Verlet integrator in TINKER. A Nosé–Hoover thermostat^59^, ^60^ was employed to constrain the temperature to 300 K with a coupling time parameter, from which the Nosé‐Hoover chain masses are set in TINKER, of 0.2 ps. Final temperature and density equilibrated structures were used as solvent box inputs for the following series of solvation free energy calculations.

### Production simulation details

AMOEBA MD simulations for solvation free energy calculations utilized either the AMBER 14^61^ or TINKER 6.3.3 packages^48^ depending on the solute/solvent system under investigation. All systems were initially prepared in TINKER^48^ by soaking each molecule in a periodic box of pre‐equilibrated solvent, generated as above, using the XYZEDIT utility of TINKER. Initial structures and parameters were then converted to AMBER format for subsequent minimization, equilibration and simulation, using the tinker_to_amber utility of AMBER 14. However, solutes or solvents that included a “Z‐Bisector” multipole local frame (DMSO, Acetonitrile, Methylamine, Trimethylamine) could not be converted as the “Z‐Bisector” frame is not implemented in AMBER 14. Instead, these simulations were performed with an equivalent procedure in TINKER 6.3.3. Details of both protocols are provided below. All simulations were performed in triplicate, using the same starting structure but a different random number seed for the thermostat.

Solution phase simulations in AMBER used the pmemd.amoeba program and were performed as follows. Initially, the systems underwent minimization for 2500 steps, of which the first 1000 steps were run with a steepest descent algorithm, and the next 1500 steps with a conjugate gradient algorithm. For each system, simulations were then performed in the NVT ensemble, heated slowly to 300 K over 50 ps, followed by another 100 ps of pressure equilibration using NPT at 300 K and 1 atm. A timestep of 1 fs and a velocity Verlet integrator was used to propagate dynamics. To maintain the temperature and pressure, the systems were treated using a Langevin thermostat and Berendsen barostat, respectively.^58^, ^62^ A different random seed for the Langevin thermostat was applied for each independent repeat simulation. van der Waals (vdW) interactions were evaluated explicitly up to a 9 Å cutoff with an analytical long‐range correction. Long‐range electrostatic interactions for all the systems were treated using a Particle Mesh Ewald (PME) summation,^63^ with a real‐space cutoff of 8 Å. The PME calculation used fifth order B‐spline interpolation. At each step the atomic induced dipoles were converged until the root‐mean square change was below 0.01 D/atom. Finally, the last configuration of the NPT simulation was used as the starting point for equilibration in all the intermediate *λ* states with AMOEBA.

A total of 11 intermediate state simulations with *λ* = 1.0, 0.9, 0.8, 0.7, 0.6, 0.5, 0.4, 0.3, 0.2, 0.1, and 0.0 were applied to electrostatic interactions for discharging the solute in vacuum and in solvent.^37^ *λ* = 1 refers to a fully interacting solute and *λ* = 0 to a noninteracting solute. However, for calculating the free energies of decoupling solute vdW interactions in the solvent, a different spacing of intermediate states was used with *λ* = 1.0, 0.9, 0.8, 0.75, 0.7, 0.65, 0.6, 0.5, 0.4, 0.2, and 0.0.^37^ Furthermore, to allow the potential to disappear smoothly as the intermediate simulations progressed to zero, a soft‐core Halgren buffered 14‐7 van der Waals term^64^ as previously described by Shi et al.^37^ was applied. For each value of *λ*, 2 ns of constant pressure molecular dynamics were performed, using an identical protocol to the NPT pressure equilibration step. Atomic coordinates of the system were saved every 1 ps and the first 200 ps of each window were discarded as equilibration.

Solution phase simulations in TINKER^48^ were performed identically to those in AMBER except for the following minor changes. Minimization in TINKER was performed using a default minimization algorithm, limited memory Broyden–Fletcher–Goldfarb–Shanno (BFGS) Quasi‐Newton optimization^65^ for 2500 steps. Additionally, the Nosé–Hoover thermostat^59^, ^60^ was employed during MD simulations instead of the Langevin thermostat^62^ of AMBER 14. All the other protocol options, including the *λ* windows applied, were identical.

All the gas phase simulations were performed in TINKER.^48^ In this simulation, a single solute molecule only was simulated for 200 ps using a stochastic integrator with a time step of 0.1 fs and a temperature of 300 K. The induced dipoles were converged to 1 × 10^−6^ D/atom. Coordinates were saved every 0.1 ps. For free energy analysis, the first 20 ps were discarded. In all case, BAR was used to evaluate the free energy changes between the neighboring states (*λ_i_* and *λ_i_*_+ 1_).

For the GAFF simulations an identical protocol was implemented except that an 8 Å direct vdW cutoff was used rather than 9 Å. Importantly, the PMEMD and SANDER modules included in AMBER 14 were used for the GAFF simulations with identical *λ* windows employed throughout for both force fields. For free energy calculations, BAR was used as implemented in the PYMBAR PYTHON package^66^ for GAFF fixed‐point‐charge results, while an in‐house script, BAR‐amber^67^ was used to analyze the results for the AMOEBA simulations.

### Statistical error analysis

The error analysis and significance testing suggested by Mobley et al.^21^ was employed to evaluate the calculated solvation free energies in four solvents simulated with both the AMOEBA and GAFF force fields. The agreement of estimated solvation free energies with experiment was evaluated using mean unsigned error (MUE), mean signed error (MSE), Pearson correlation coefficient (R), coefficient of determination (*R* ^2^) and Kendall's tau coefficient (*τ*) across three replicates. In addition, 1000 iterations of bootstrapping with replacement were performed to estimate the 95% confidence intervals on these values. Finally, a Student's paired *t*‐test was applied to determine the significance of differences between MSE errors generated with AMOEBA and GAFF assuming both are normally distributed. A Wilcoxon signed‐rank test was used to similarly compare MUE since they are severely non‐normally distributed. These tests will indicate whether the errors of our predictions are substantially different between different force fields.

## Results and Discussion

### Solvent comparison

**Figure 3:**
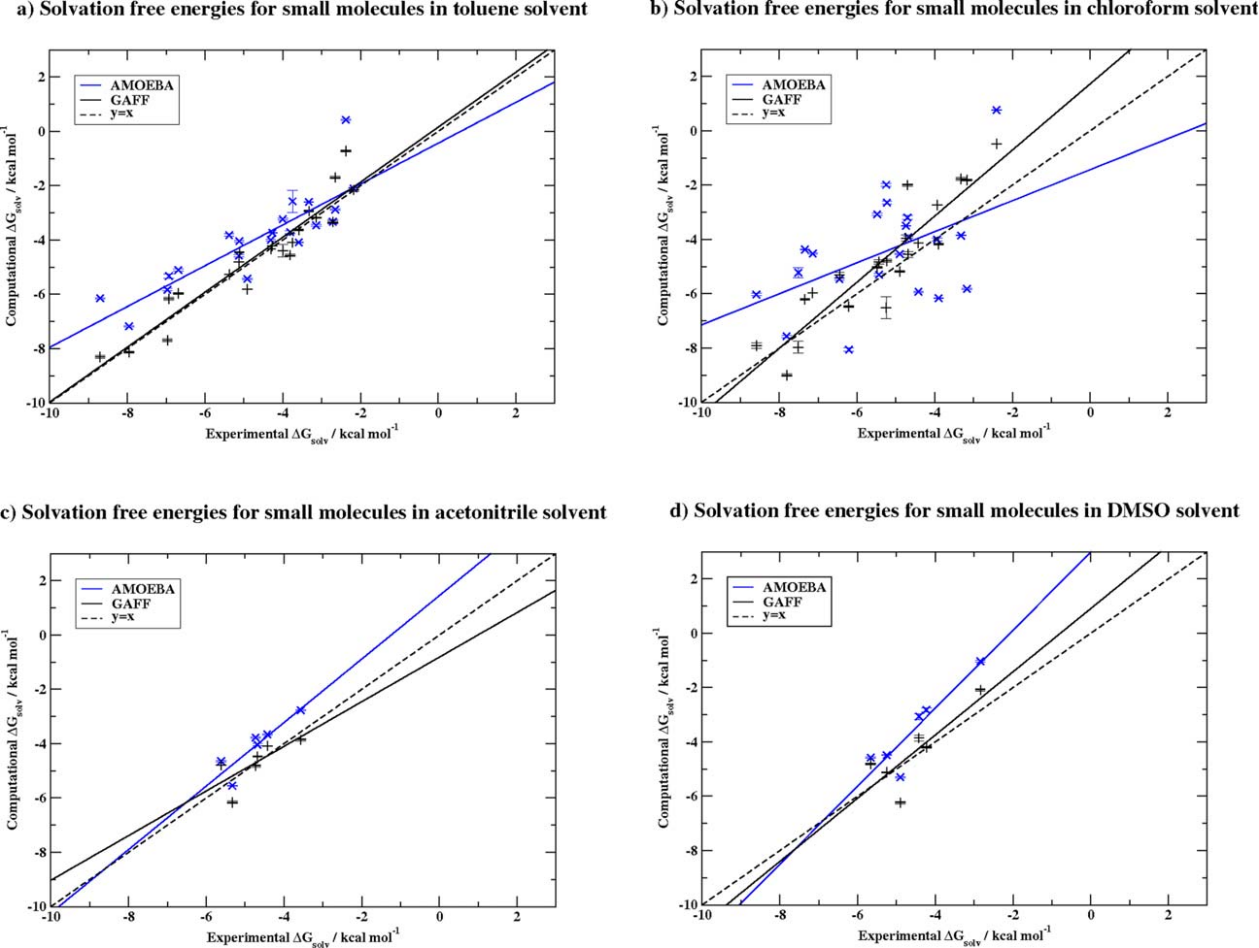
AMOEBA (blue) and GAFF (black) calculated Δ*G*_solv_ for small molecules in toluene, chloroform, acetonitrile and DMSO against experimental Δ*G*_solv_. Line of perfect agreement, *y* = *x*, shown as dashed line. Linear regression in each solvent plot gives the following equations: a) AMOEBA (*y* = 0.752 *x* − 0.4375), GAFF (*y* = 1.012 *x* + 0.153) b) AMOEBA (*y* = 0.571 *x* − 1.435), GAFF (*y* = 1.217 *x* + 1.722) c) AMOEBA (*y* = 1.169 *x* + 1.452), GAFF (y = 0.822 *x* − 0.813) and d) AMOEBA (*y* = 1.436 *x* + 2.986), GAFF (*y* = 1.164 *x* + 0.907). [Color figure can be viewed at wileyonlinelibrary.com]

The calculated solvation free energies of each solute in all four solvents, with the associated standard error and unsigned error to experiment, are provided in Table S1–S4 in the Supporting Information. The error in each estimated value of $\;\Delta G_{\text{solv}\;}$ corresponds to the standard error in the mean across three repeats. The small standard error for AMOEBA and GAFF simulations in all datasets (∼0.1 kcal mol^−1^) provides no evidence to indicate inadequate conformational sampling and hence we assessed the simulations to be of appropriate length. Figure 3 compares AMOEBA and GAFF solvation free energy results across all four solvents directly with those of experiment, while Table 1 provides summary metrics of the same results.

**Table 1:** Summary of performance metrics for calculated solvation free energies with the AMOEBA polarizable force field and the GAFF fixed‐point‐charge force field in all four solvents.

	Solvent			
Metrics	Toluene	Chloroform	Acetonitrile	DMSO
**AMOEBA force field**				
MUE (kcal mol^−1^)	0.67 ≤ **0.92** ≤1.30	1.23 ≤ **1.68** ≤ 2.09	0.48 ≤ **0.73** ≤ 0.88	0.74 ≤ **1.12** ≤ 1.46
MSE (kcal mol^−1^)	0.37 ≤ **0.73** ≤ 1.14	0.12 ≤ **0.90** ≤ 1.57	0.10 ≤ **0.65** ≤ 0.88	0.20 ≤ **0.99** ≤ 1.4
R	0.74 ≤ **0.86** ≤ 0.92	0.18 ≤ **0.51** ≤ 0.79	−1.00 ≤ **0.89** ≤ 0.99	−0.63 ≤ **0.91** ≤ 1.00
*R* ^2^	0.53 ≤ **0.74** ≤ 0.85	0.03 ≤ **0.26** ≤ 0.62	0.15 ≤ **0.79** ≤ 0.97	0.17 ≤ **0.84** ≤ 1.00
Kendall *τ*	0.53 ≤ **0.74** ≤ 0.88	−0.12 ≤ **0.23** ≤ 0.51	0.33 ≤ **0.73** ≤ 1.00	−0.09 ≤ **0.73** ≤ 1.00
**GAFF Force Field**				
MUE (kcal mol^−1^)	0.32 ≤ **0.48** ≤ 0.68	0.68 ≤ **0.92** ≤ 1.23	0.21 ≤ **0.43** ≤ 0.67	0.27 ≤ **0.61** ≤ 0.98
MSE (kcal mol^−1^)	−0.14 ≤ **0.10** ≤ 0.40	0.18 ≤ **0.56** ≤ 1.01	−0.44 ≤ **0.03** ≤ 0.41	−0.68 ≤ **0.16** ≤ 0.58
R	0.89 ≤ **0.95** ≤ 0.98	0.78 ≤ **0.91** ≤ 0.96	−1.00 ≤ **0.73** ≤ 0.93	−0.05 ≤ **0.82** ≤ 0.99
*R* ^2^	0.80 ≤ **0.90** ≤ 0.95	0.60 ≤ **0.83** ≤ 0.92	0.00 ≤ **0.53** ≤ 0.85	0.00 ≤ **0.68** ≤ 0.97
Kendall *τ*	0.72 ≤ **0.87** ≤ 0.96	0.59 ≤ **0.77** ≤ 0.88	−0.09 ≤ **0.73** ≤ 1.00	−0.23 ≤ **0.47** ≤ 1.00

Upper and lower bounds estimated as 95% confidence intervals in the mean using bootstrapping for 1000 iterations with replacement.

The mean unsigned error to experiment for calculated solvation free energies across all solvents is approximately 1.22 kcal mol^−1^ for AMOEBA and 0.66 kcal mol^−1^ for GAFF (Supporting Information Tables S1–S4). The largest MUE is in chloroform solvent for both force fields as shown in Table 1. In terms of MSE both force fields underestimate solvation free energies (i.e., show positive MSE) particularly for the ammonia solute simulated with AMOEBA in toluene and chloroform (Supporting Information Tables S1 and S2). Predominantly, the AMOEBA MSE in all solvents is slightly larger than that of GAFF, as shown in Table 1.

Interestingly, the results of solvation free energies with GAFF often give better correlation to experimental data based on comparison of the four solvents in Figure 3 and Table 1. The best agreement was given in toluene with *R* ^2^ 0.90 (Fig. 3a) while the worst *R* ^2^ of 0.53 was observed in acetonitrile (Fig. 3d). Similarly to the MUE metrics above, chloroform solvation free energies for small molecules using AMOEBA showed the worst correlation to experimental values with *R* ^2^ 0.26 (Fig. 3c). The best *R* ^2^ for AMOEBA of 0.84 was in DMSO, and may be due to a consistent underestimation of solvation free energy calculated across the whole dataset, as suggested by the linear regression line observed in Figure 3d.

To allow performance comparison of AMOEBA and GAFF in different environments, the results of each solvent were also compared using their Kendall *τ* coefficients, which examined agreement in ranking of solvation free energies between theory and experiment. Kendall *τ* allowed the determination of a clear order of performance for all solvents in the two different force fields. With AMOEBA, toluene, DMSO and acetonitrile perform well (overall *τ* values of 0.74, 0.73, and 0.73, respectively), while chloroform again performs worst with only 0.23. For GAFF, *τ* value for toluene indicates the best agreement in predicted rankings (*τ* = 0.87) followed by chloroform (0.77), acetonitrile (0.73), and DMSO (0.47). It should be borne in mind that the small dataset sizes for acetonitrile and toluene (*n* = 6) may lead to large fluctuations in *τ* and *R* with small changes in results, as demonstrated by the broader confidence intervals for these measures.

### Statistical error analysis

Since the available experimental dataset is very small, it is difficult to assess the performance of the force field based on the comparison of mean metrics. Statistical confidence intervals estimated via bootstrapping allow a more relevant comparison between metrics to be made. Bootstrapping with replacement was performed for 1000 iterations, and the 95% confidence intervals in all metrics were calculated from the underlying distributions. Additionally a Student's paired *t*‐test and Wilcoxon signed‐rank test were performed using the original signed and unsigned error distributions (respectively) for AMOEBA and GAFF, to assess whether differences between force fields were statistically significant. Table 1 shows the ranges in these metrics computed in all solvents for both AMOEBA and GAFF. Looking at the results for MUE and MSE of solvation free energies provided in all solvents, the magnitude of the associated ranges remains similar between AMOEBA and GAFF, suggesting that the performance across solvents is consistent in terms of error.

Regarding the *t*‐test and Wilcoxon signed‐rank test results (Table 2), evaluating AMOEBA and GAFF differences in MSE and MUE, there is a significant difference between AMOEBA and GAFF MSE for all solvents except chloroform (significance threshold of *p* = 0.05). However, analysis of MUE distributions showed significant differences only in chloroform and toluene. DMSO and acetonitrile yield no significant difference between their very similar range of MUE.

**Table 2:** Calculated *p*‐values of statistical tests between mean signed (Student's paired *t*‐test) and unsigned error (Wilcoxon signed‐ranked test) distributions for AMOEBA and GAFF.

	Solvent			
*p*‐Value	Toluene	Chloroform	Acetonitrile	DMSO
Unsigned Error	**0.0071**	**0.0087**	0.2489	0.1730
Signed Error	**0.0015**	0.4363	**0.0098**	**0.0028**

Significant differences (*p* < 0.05) denoted in bold. GAFF and AMOEBA perform identically in terms of MUE for acetonitrile and DMSO, and in terms of MSE in chloroform. For all other metrics GAFF performed better.

### Analysis of the performance

Generally, the examination of results in Table 1 reveals that overall the AMOEBA polarizable force field performs well, but slightly worse than GAFF when compared to the experimental data. There are a number of molecules found to give the largest errors to experiment across all the solvents. Ammonia has a consistently underestimated (too positive) Δ*G*_solv_ in both solvents for which its solvation free energy was evaluated. This trend was observed for both AMOEBA and GAFF force fields, suggesting a non‐potential‐specific systematic error. This may therefore suggest a doubt in the accuracy of the experimental data. The experimental free energies of solvation for our solutes were calculated in one of two ways: (i) using direct partition coefficients between gas phase and liquid phase, or (ii) using partition coefficients between water and nonaqueous solvents, combined with hydration free energies. However, predominantly the latter approach was used—experimental measurements were determined by combining both experimental values for aqueous hydration free energies and partition coefficients measured between water and nonaqueous liquids.^40^ The average uncertainty in experimental values of solvation free energies reported by the authors of the Minnesota solvation database is ∼ 0.2 kcal mol^−1^ for the subset used in this study.^40^, ^68^, ^69^ However, this uncertainty is likely to be non‐normally distributed amongst the members of the database, such that individual molecules may have larger or smaller errors in their experimental Δ*G* estimates. The experimental errors for specific molecules are not provided by the Minnesota solvation database, but the consistently poor performance of a molecule across solvents and force fields studied, such as in the case of ammonia, may suggest a larger than average experimental error for that solute.

Apart from this, one of the areas that may have an impact on the accuracy of solvation free energy calculation is the parameterization. Both solute and solvents need to be well parameterized to give the correct solvation free energy estimates. For AMOEBA, we have shown elsewhere how small changes in parameterization methodology can give significant differences in hydration free energies.^38^ However, owing to the simplicity of the molecules constituting the dataset used here, it is difficult to introduce further systematic modifications to the solute parameterization protocol without fundamental change to the underlying parameterization philosophy (e.g., by fitting to solvent–solute interaction energies). Our aim here has been to follow the optimum AMOEBA parameterization protocols closely. In particular, multipole coordinate frames and polarization groups were manually defined, valence parameters were taken from the established amoeba09 parameter set, and atomic multipoles were fitted to molecular ESP calculated using the recommended large basis set (aug‐cc‐pVTZ). Thus, parameterization on the whole was performed as per well‐established guidelines.^37^, ^38^, ^39^

There may also be occasions where parameterization remains challenging. In our case, the largest errors to experiment for AMOEBA solvation free energy predictions are mostly from ammonia, *n*‐octane and hexanoic acid molecules. The simplest of the molecules studied, such as ammonia, may be highly sensitive to small parameter changes. If the potential of each atom interacting with the solvent is even slightly overestimated, this may contribute to the significant overestimation of the solvation free energies for ammonia in chloroform and toluene. Additionally, parameters for *n*‐octane or hexanoic acid may be affected by the conformation or conformations used in the multipole generation process. For these molecules with extended chains there are many conformations that are low in energy and visited during the MD simulation. It is challenging to select the correct low energy conformation for multipole assignment for those molecules. Unlike other studies, we did not attempt to include multiple conformations in the ESP fitting process, as the majority of molecules studied had single, fairly rigid, well‐defined low energy conformations. Beyond solute parameters, as results for small molecules in chloroform were consistently the worst compared to other solvents, the AMOEBA solvent models also needed to be considered. Liquid phase tests do exist in the paper describing the chloroform potential, including density, and heat of vaporization, but they are fairly simple.^42^ These properties have also been evaluated for other solvent models used here.^39^, ^45^, ^46^, ^47^ Nevertheless, it should be noted that these measures only validate solvent‐solvent interactions and do not assess the accuracy of solute–solvent interactions, as would be necessary for accuracy in our solvation free energy calculations.

Sampling is also a common issue when running molecular dynamics simulations. Considering the molecules are fairly small, it is not surprising that they quickly converge, as demonstrated by the small uncertainties observed for the majority of molecules. Notably *n*‐octane and hexanoic acid may be exceptions to this rule, as demonstrated by the higher than average standard errors observed in their estimates, particularly in chloroform (Supporting Information Table S2). The sampling of different conformations to reach equilibrium may have been problematic during the short timescales simulated here. However, variance in estimates due to differential sampling between repeats did not increase the error systematically between solvents. Moreover the increased uncertainty in predictions it caused was not the predominant driver of poor agreement in chloroform, where other solutes had equal or greater error to experiment.

As noted above, GAFF typically performed well for most functional groups with better accuracy to experiment compared to AMOEBA. This improvement spanned both polar and nonpolar solvents, and solutes containing a multitude of functional groups. There was also no clear consistency in observed errors for particular solute functional groups; however, given its size, the current dataset is limited in its ability to discern trends in functional groups. The largest functional group subset, amines, consisted of five compounds (Ammonia, aniline, methylamine, diethylamine, and trimethylamine), for which experimental data was only available in the nonpolar solvents toluene and chloroform. An extended study on a broader dataset would be required to investigate functional group trends further.

The solvent models used in fixed‐point‐charge simulations with GAFF solute parameters had not been optimized for solvation free energy calculations during their respective parameterizations.^45^, ^46^, ^47^ It is somewhat surprising, therefore, that all solvents showed consistently reasonable agreement with experiment. In general therefore, these results may suggest that explicit electronic polarization may not be crucial for good agreement with experiment. Here, all nonaqueous solvents investigated have dielectric constants smaller than water. In this type of environment, the effect of molecular polarization on solvation free energies may be less, and a fixed‐point‐charge model of electrostatic interaction may be sufficient. Evaluation with a nonaqueous solvent with higher dielectric constant than water, such as formamide (dielectric constant = 111),^43^ would provide further information on the effect of an explicit treatment of polarization in different environments. However, computational nonaqueous solvation free energy studies are hampered by the scarcity of suitable experimental data for multiple solutes across multiple solvents. Therefore, while it remains unknown if an explicit representation of polarization may be required for accuracy in more complex electrostatic environments, the simple systems studied here perhaps are better represented by simple force fields rather than a thorough application of polarization terms as incorporated in the AMOEBA force field.

## Conclusions

Overall, both force fields estimated nonaqueous solvation free energies well, with only the AMOEBA chloroform and DMSO results exhibiting MUE above the 1.0 kcal mol^−1^ limit often considered as “chemical accuracy” in free energy calculations. Our findings that chloroform solvation free energies have the largest errors to experiment, despite reasonable correlation for GAFF, are consistent with the recent results of Zhang et al.^36^

GAFF showed statistically significant improvements in unsigned error over AMOEBA for the 21‐solute datasets of toluene and chloroform, and in signed error for all but chloroform. This improvement is likely a combination of two factors. First, the GAFF force field, first established in 2004,^32^ is now a well‐developed and well understood small molecule force field, whose solute parameters (beyond the independently derived point charges) have undergone multiple rounds of refinement and been used in multiple other free energy investigations and blind challenges.^21^, ^27^, ^31^, ^70^, ^71^ This extensive history of testing and development is clearly beneficial for GAFF performance, as demonstrated here and in the other recent solvation free energy studies described above. In contrast AMOEBA parameters, both solvent and solute, have not been tested as extensively or empirically adjusted to recreate thermodynamic properties. This is particularly highlighted by the relatively poor AMOEBA performance in chloroform. While AMOEBA parameters provide an excellent description of the electrostatic environment surrounding the chloroalkanes, including σ‐hole effects, bulk phase thermodynamic properties were not included as targets in the parameter optimization process.^42^

Second, as discussed, the low dielectric solvents (and often simple solutes) tested here may not require the additional rigor of a polarizable force field for accurate free energy estimates. AMOEBA has previously been shown to recreate instantaneous fluctuations in electric fields in nonpolar solvents, and the resulting shifts in the vibrational spectroscopy of probe groups, far more accurately than a fixed‐charge model.^72^ Nevertheless, from our work it appears that the simpler electrostatics representation used by GAFF and many other force fields may be sufficient for standard thermodynamic metrics (such as solvation free energies) in low polarity environments.

Evaluation of more challenging solutes and solvents is, however, extremely limited by a lack of relevant experimental data for comparison. The Minnesota solvation database is a well‐curated resource and has been used in the development of multiple solvation schemes. However, its dataset of > 3000 entries does not include any solvation free energies in solvents of higher dielectric than water.^40^ Additionally, only a limited number of neutral solutes have their solvation free energies measured in multiple solvents. These difficulties in the curating of solvation free energies for force field evaluation are well known and have led to the use of alternate metrics with more abundant experimental data, such as solubility or distribution coefficient calculations, in recent tests.^73^, ^74^ These metrics provide promising ways of evaluating multiple protocols in blind tests, but, as demonstrated here, there remains a role for computationally more straightforward absolute free energy calculations. Despite these challenges, our broad comparison of potential functions across a range of systems identifies clear opportunities for force field improvements, and we believe further work should ideally focus on the context of high field environments, where requirements for polarization may be more apparent.

## Supporting information

Supporting Information
