# Supplementary material for: Evaluation of solvation free energies for small molecules with the AMOEBA polarizable force field
Source: J Comput Chem. 2016 Oct 19;37(32):2749–58. doi: 10.1002/jcc.24500 (PMC5111595; doi:10.1002/jcc.24500)
Supplement: Supplementary file 1 — Supporting Information [file JCC-37-2749-s001.pdf]

Supporting information

Evaluation of solvation free energies for small molecules with the AMOEBA polarizable force field

Noor Asidah Mohamed[1], Richard T. Bradshaw[1] and Jonathan W. Essex[1]

[1]School of Chemistry, University of Southampton, Highfield, Southampton, SO17 1BJ

Correspondence to: Jonathan W. Essex (E-mail: *j.w.essex@soton.ac.uk*)

Table S1. AMOEBA calculated solvation free energies for small molecules in toluene (ε = 2.38) against experimental data and fixed-point charge (GAFF) data.

	ΔG_{tol} (kcal mol^{-1})			Unsigned Error to Experiment	
Molecule	Exp	AMOEBA	GAFF	AMOEBA	GAFF
1,4-dioxane	-4.91[a]	-5.43 ± 0.02	-5.82 ± 0.00	0.52	0.91
2-butanone	-4.27	-3.74 ± 0.01	-4.22 ± 0.02	0.53	0.05
Acetic acid	-4.00	-3.24 ± 0.04	-4.39 ± 0.34	0.76	0.39
Acetone	-3.59	-4.09 ± 0.01	-3.63 ± 0.03	0.50	0.04
Ammonia	-2.38	0.42 ± 0.03	-0.73 ± 0.03	2.80	1.65
Aniline	-6.69	-5.11 ± 0.04	-5.97 ± 0.03	1.58	0.72
Ethanol	-3.33	-2.60 ± 0.01	-2.94 ± 0.03	0.73	0.39
Methanol	-2.18	-2.10 ± 0.04	-2.16 ± 0.06	0.08	0.02
Methylamine	-2.65	-2.88 ± 0.03	-1.70 ± 0.04	0.23	0.95
n-octane	-5.38	-3.82 ± 0.04	-5.27 ± 0.01	1.56	0.11
Nitromethane	-4.31	-4.00 ± 0.03	-4.34 ± 0.01	0.31	0.03
Phenol	-6.93	-5.33 ± 0.08	-6.17 ± 0.06	1.60	0.76
Pyridine	-5.13	-4.58 ± 0.04	-4.81 ± 0.02	0.55	0.32
Toluene	-5.12	-4.04 ± 0.03	-4.45 ± 0.03	1.08	0.67
Diethylamine	-3.75	-2.58 ± 0.40	-4.10 ± 0.01	1.17	0.35
P-bromophenol	-8.70	-6.15 ± 0.02	-8.31 ± 0.05	2.55	0.39
Trimethylamine	-2.71	-3.33 ± 0.08	-3.34 ± 0.04	0.62	0.63
Hexanoic acid	-6.97	-5.83 ± 0.04	-7.69 ± 0.06	1.14	0.72
Methylacetate	-3.81	-3.72 ± 0.06	-4.56 ± 0.04	0.09	0.75
Methylbenzoate	-7.96	-7.18 ± 0.03	-8.13 ± 0.03	0.78	0.17
Hydrogen peroxide	-3.14	-3.34± 0.05	-3.18 ± 0.03	0.20	0.04

All the experimental solvation free energies are taken from the Minnesota solvation database[1] except [a]Experimental solvation free energies taken from Abraham *et al.*[2] Errors report 1 standard error over 3 repeats.

Table S2. AMOEBA calculated solvation free energies for small molecules in chloroform (ε = 4.81) against experimental data and fixed-point charge (GAFF) data.

		ΔG_{chl} (kcal mol^{-1})		Unsigned Error to Experiment	
Molecule	Exp.	AMOEBA	GAFF	AMOEBA	GAFF
1,4-dioxane	-6.21[a]	-8.06 ± 0.06	-6.47 ± 0.04	1.85	0.38
2-butanone	-5.43	-5.30 ± 0.05	-4.83 ± 0.07	0.13	0.60
Acetic acid	-4.74	-3.50 ± 0.01	-3.95 ± 0.11	1.24	0.79
Acetone	-4.42	-5.93 ± 0.02	-4.14 ± 0.01	1.51	0.28
Ammonia	-2.41	0.76 ± 0.01	-0.49 ± 0.01	3.17	1.92
Aniline	-7.34	-4.37 ± 0.01	-6.21 ± 0.03	2.97	1.13
Ethanol	-3.94	-4.00 ± 0.05	-2.74 ± 0.01	0.06	1.20
Methanol	-3.32	-3.86 ± 0.01	-1.78 ± 0.05	0.54	1.54
Methylamine	3.17	-5.14 ± 0.01	-1.82 ± 0.01	1.97	1.35
n-octane	-5.25	-1.99 ± 0.05	-6.52 ± 0.41	3.26	1.27
Nitromethane	-4.68	-3.92 + 0.03	-4.55 ± 0.11	0.76	0.13
Phenol	-7.14	-4.52 ± 0.02	-5.97 ± 0.02	2.62	1.17
Pyridine	-6.45	-5.46 ± 0.03	-5.31 ± 0.10	0.99	1.14
Toluene	-5.48	-3.08 ± 0.04	-5.03 ± 0.03	2.40	0.45
Diethylamine	-5.23	-2.65 ± 0.05	-4.79 ± 0.05	2.58	0.44
P-bromophenol	-8.59	-6.03 ± 0.04	-7.91 ± 0.10	2.56	0.68
Trimethylamine	-3.90	-6.17 ± 0.01	-4.18 ± 0.04	2.27	0.28
Hexanoic acid	-7.51	-5.23 ± 0.18	-7.97 ± 0.21	2.28	0.46
Methylacetate	-4.90	-4.54 ± 0.03	-5.18 ± 0.04	0.36	0.28
Methylbenzoate	-7.81	-7.57 ± 0.04	-9.00 ± 0.04	0.24	1.19
Hydrogen peroxide	-4.70	-3.20 ± 0.05	-2.00 ± 0.04	1.50	2.70

All the experimental solvation free energies are taken from the Minnesota solvation database[1] except [a]Experimental solvation free energies taken from Abraham *et al.*[2] Errors report 1 standard error over 3 repeats.

Table S3. AMOEBA calculated solvation free energies for small molecules in acetonitrile (ε = 36.64) against experimental data and fixed-point charge (GAFF) data.

	ΔG_{ace} (kcal mol^{-1})			Unsigned Error to Experiment	
Molecule	Exp	AMOEBA	GAFF	AMOEBA	GAFF
1,4-dioxane	-5.33[a]	-5.55 ± 0.01	-6.16 ± 0.04	0.22	0.83
2-butanone	-4.73	-3.78 ± 0.02	-4.82 ± 0.04	0.95	0.09
Ethanol	-4.43	-3.66 ± 0.02	-4.08 ± 0.01	0.77	0.36
n-octane	-3.57	-2.77 ± 0.02	-3.86 ± 0.05	0.80	0.29
Nitromethane	-5.62	-4.64 ± 0.02	-4.79 ± 0.02	0.98	0.83
Toluene	-4.68	-4.04 ± 0.04	-4.47 ± 0.03	0.64	0.21

All the experimental solvation free energies are taken from Minnesota solvation database[1] except [a]Experimental solvation free energies taken from Abraham *et al.*[2] Errors report 1 standard error over 3 repeats.

Table S4. AMOEBA calculated solvation free energies for small molecules in DMSO (ε = 47.24) against experimental data and fixed-point charge (GAFF) data.

	ΔG_{dmso} (kcal mol^{-1})			Unsigned Error to Experiment	
Molecule	Exp	AMOEBA	GAFF	AMOEBA	GAFF
1,4-dioxane	-4.90[a]	-5.27 ± 0.03	-6.24 ± 0.04	0.37	1.34
2-butanone	-4.23	-2.87 ± 0.07	-4.20 ± 0.03	1.36	0.03
Ethanol	-5.25	-4.48 ± 0.01	-5.12 ± 0.02	0.77	0.13
n-octane	-2.84	-1.04 ± 0.04	-2.09 ± 0.05	1.80	0.75
Nitromethane	-5.66	-4.56 ± 0.02	-4.81 ± 0.03	1.10	0.85
Toluene	-4.42	-3.09 ± 0.11	-3.86 ± 0.11	1.33	0.56

All the experimental solvation free energies are taken from Minnesota solvation database[1] except [a]Experimental solvation free energies taken from Abraham *et al.*[2] Errors report 1 standard error over 3 repeats.

References

(1) Marenich, A. V.; Kelly, C. P.; Thompson, J. D.; Hawkins, G. D.; Chambers, C. C.; Giesen, D. J.; Winget, P.; Cramer, C. J.; Truhlar, D. G. Minnesota Solvation Database-version 2012 http://comp.chem.umn.edu/mnsol/ (accessed Jul 29, 2016).

(2) Abraham, M. H.; Platts, J. A.; Hersey, A.; Leo, A. J.; Taft, R. W. *J. Pharm. Sci.* **1999**, *88* (7), 670–679.
